# A Case of Diffuse Large B Cell Lymphoma With Rapidly Developing Abdominal Distension As the Only First Clinical Presentation

**DOI:** 10.7759/cureus.64837

**Published:** 2024-07-18

**Authors:** Xiaoda Song, Yuchen Wei, Ning Zhang, Xiaohong Sun, Lin Kang

**Affiliations:** 1 Department of Internal Medicine, Peking Union Medical College Hospital, Chinese Academy of Medical Sciences, Beijing, CHN; 2 Department of Geriatrics, Peking Union Medical College Hospital, Chinese Academy of Medical Sciences, Beijing, CHN

**Keywords:** hepatitis b virus, older patients, a case, diagnosis, diffuse large b-cell lymphoma

## Abstract

This article presents a case study of diffuse large B-cell lymphoma in an elderly patient whose initial clinical manifestation was rapidly developing abdominal distension. The article delves into the patient's diagnostic and treatment journey, highlights treatment insights, and reviews relevant literature. The aim is to enhance the clinical diagnosis accuracy for elderly lymphoma patients presenting with a singular atypical symptom, ultimately optimizing treatment plans and enriching clinicians' knowledge of the disease.

## Introduction

Diffuse large B-cell lymphoma (DLBCL) is a highly aggressive form of non-Hodgkin's lymphoma (NHL), accounting for 30% to 40% of all NHL cases. The incidence of DLBCL increases with age [1,2]. Clinically, most patients present with a rapidly growing mass affecting one or more lymph nodes and extranodal sites, often accompanied by systemic symptoms such as anemia, fever, and weight loss, as well as local symptoms like dyspnea and hepatosplenomegaly [1,3]. Additionally, serum lactate dehydrogenase (LDH) levels are typically elevated [1]. Early symptoms of DLBCL are often subtle, and advanced stages can result in widespread multi-organ involvement that is life-threatening [3]. Therefore, early diagnosis is crucial to improve patient outcomes. This article presents a case study of DLBCL with rapidly progressive abdominal distension as the initial clinical sign, along with a literature review to enhance clinicians' knowledge of this disease.

## Case presentation

**Figure 1:**
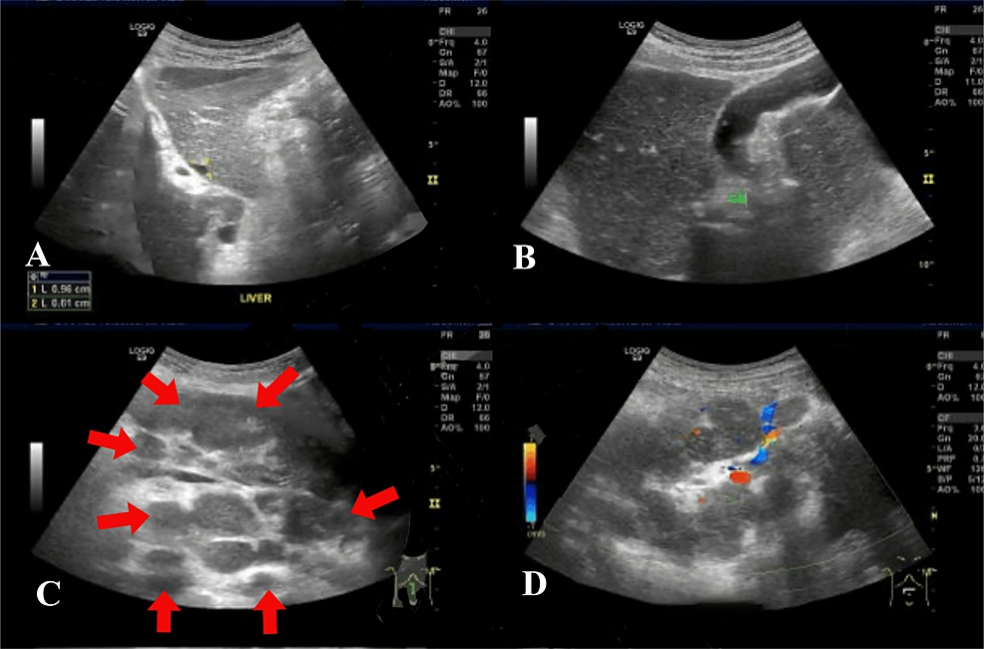
Abdominal ultrasound FIGURE 1A and 1B and 1C and 1D: Abdominal ultrasound revealed a hypoechoic mass in the abdominal cavity extending from below the pancreas to the level of the umbilicus, measuring approximately 17.3×15.3×8.7cm, with an irregular shape and unclear boundaries, appearing to be composed of multiple hypoechoic areas fused together.

The patient, a 60-year-old elderly male, was admitted to the Geriatrics Department of Peking Union Medical College Hospital on April 30, 2024, presenting with abdominal distension that had persisted for three weeks. The abdominal distension began after the patient took 500 mg of metformin three times a day for three days in mid-April 2024, and was accompanied by intermittent acid reflux and belching. The patient described a feeling of abdominal distension and hardness, along with occasional dull pain around the umbilicus in the morning before going to bed. The patient rated the pain on the Numeric Rating Scale (NRS) as 2-3, and reported that it would subside on its own after a few minutes. There were no abdominal cramps, bloody stools, melena, lower limb edema, shortness of breath, or exertional dyspnea. An abdominal ultrasound on April 26 revealed a hypoechoic mass in the abdominal cavity extending from below the pancreas to the level of the umbilicus, measuring approximately 17.3×15.3×8.7cm, with an irregular shape and unclear boundaries, appearing to be composed of multiple hypoechoic areas fused together (Figure 1). Additionally, a free fluid collection was noted in the lower abdomen, approximately 4.3cm deep. Throughout the illness, the patient did not experience fever, night sweats, nausea, vomiting, drowsiness, gross hematuria, or muscle spasms; however, he did report occasional skin itching. The patient's mental health and sleep were reported as good, with a frequency of nocturia of two times per night, bowel movements occurring one to two times per day, and passing yellow, mushy stools. Due to the abdominal distension, the patient had reduced food intake, resulting in a weight loss of 3.5kg over a period of two months.

The patient's medical history includes a diagnosis of type 2 diabetes in 2010 with a blood glucose level of 16mmol/L. Insulin aspart and oral acarbose were initially prescribed for glucose control, with subsequent adjustment to metformin and repaglinide in 2024. However, metformin was discontinued due to abdominal distension. In 2018, elevated blood pressure was noted and managed with amlodipine. Additionally, the patient was found to have hepatitis B in 2010 but was asymptomatic. A long history of smoking, with recent cessation, is reported while denying any alcohol consumption.

Upon admission, the patient exhibited normal vital signs and physical characteristics, with a body temperature of 36.0℃, heart rate of 69 beats/min, respiratory rate of 17 beats/min, SpO2 of 95%, blood pressure of 139/85mmHg, height of 164cm, body weight of 63kg, BMI of 23.4kg/㎡, and an abdominal circumference of 101cm. During examination, two oval-shaped lymph nodes were palpable in the left inguinal area, measuring approximately 1.5 cm long in diameter, with a tough texture, moderate mobility, and no adhesion or tenderness. Other superficial lymph nodes were not enlarged. The patient's heart rhythm was regular, breath sounds in both lower lungs were diminished, and no abnormal lung sounds were detected. Abdominal examination revealed distension without peristaltic waves or varicose veins, with dullness upon percussion and a palpable hard mass extending from the left to right of the umbilicus, displaying irregular shape and poor mobility. No tenderness, rebound tenderness, or muscle tension was noted in the abdomen. The liver and spleen were not palpable, and there was no percussion pain in the liver or kidney area. Bowel sounds were present, and shifting dullness was positive. The patient had no lower limb edema. Nutritional assessment using the Nutritional Risk Screening (NRS-2002) yielded a score of 3 points, indicating nutritional risk.

Laboratory tests were as follows: blood routine: RBC 5.14×1012/L (normal range: 4.00-5.50×1012/L), HBG 143g/L (normal range: 120-160g/L), platelet count (PLT) 258×109/L (normal range: 100-350×109/L), WBC 6.75×109/L (normal range: 3.50-9.50×109/L), lymphocyte ratio (LY%) 17.6% (normal range: 20.0-40.0%), neutrophil ratio (NEUT%) 62.6% (normal range: 50.0-75.0%), eosinophil ratio (EOS%) 12.3% (normal range: 0.5-5.0%), eosinophil count (EOS#) 0.83×109/L (normal range: 0.02-0.50×109/L). Blood biochemistry: K 4.0mmol/L (normal range: 3.5-5.5mmol/L), Mg 0.94mmol/L (normal range: 0.70-1.10mmol/L), Na 142mmol/L (normal range: 135-145mmol/L), Cl 107mmol/L (normal range: 96-111mmol/L), Ca 2.35mmol/L (normal range: 2.13-2.70mmol/L), Cr 54umol/L (normal range: 59-104mmol/L), Alb 44g/L (normal range: 35-52g/L), TBil 8.7 umol/L (normal range: 5.1-22.2umol/L), DBil 3.4umol/L (normal range: ≤6.8umol/L), urea 4.54mmol/L (normal range: 2.78-7.14mmol/L), alanine aminotransferase (ALT) 11U/L (normal range: 9-50U/L), Glu 3.7mmol/L (normal range: 3.9-6.1mmol/L); arterial blood gas analysis: pH 7.39 (normal range: 7.35-7.45), pCO2 45mmHg (normal range: 35-45mmHg), pO2 80mmHg (normal range: 83-108mmHg), Lac 1.0mmol/L (normal range: 0.5-1.7mmol/L). Coagulation: prothrombin time (PT) 11.6s (normal range: 10.4-12.6s), activated partial thromboplastin time (APTT) 26.0s (normal range: 23.3-32.5s), D-Dimer 0.76mg/L (normal range: 0-0.55mg/L); hypersensitive C-reactive protein (hsCRP) 2.10mg/L (normal range: ≤8.00mg/L); tumor necrosis factor-α (TNF-α) 86.7pg/ml (normal range: <8.1pg/ml); erythrocyte sedimentation rate (ESR) 3mm/h (normal range: 0-15mm/h); hepatitis B virus - deoxyribonucleic acid (HBV - DNA) quantification: HBV-DNA <1×103 copies/ml (normal range: <1×103 copies/ml; Table 1).

**Table 1:** Laboratory tests PLT - platelet count; LY% - lymphocyte ratio; NEUT% - neutrophil ratio; EOS% - eosinophil ratio; EOS# - eosinophil count; PT - prothrombin time; APTT - activated partial thromboplastin time; ALT - alanine aminotransferase; hsCRP - hypersensitive C-reactive protein; TNF-α - tumor necrosis factor-α; ESR - erythrocyte sedimentation rate; HBV-DNA -  hepatitis B virus - deoxyribonucleic acid; CDFI - color doppler flow imaging; MPO - myeloperoxidase

Category	Test	Result	Normal range
Blood routine	RBC	5.14×10^12/L	4.00-5.50×10^12/L
	HBG	143g/L	120-160g/L
	PLT	258×10^9/L	100-350×10^9/L
	WBC	6.75×10^9/L	3.50-9.50×10^9/L
	LY%	17.60%	20.0-40.0%
	NEUT%	62.60%	50.0-75.0%
	EOS%	12.30%	0.5-5.0%
	EOS#	0.83×10^9/L	0.02-0.50×10^9/L
Blood biochemistry	K	4.0mmol/L	3.5-5.5mmol/L
	Mg	0.94mmol/L	0.70-1.10mmol/L
	Na	142mmol/L	135-145mmol/L
	Cl	107mmol/L	96-111mmol/L
	Ca	2.35mmol/L	2.13-2.70mmol/L
	Cr	54umol/L	59-104umol/L
	Alb	44g/L	35-52g/L
	TBil	8.7umol/L	5.1-22.2umol/L
	DBil	3.4umol/L	≤6.8umol/L
	Urea	4.54mmol/L	2.78-7.14mmol/L
	ALT	11U/L	9-50U/L
	Glu	3.7mmol/L	3.9-6.1mmol/L
Arterial blood gas	pH	7.39	7.35-7.45
	pCO2	45mmHg	35-45mmHg
	pO2	80mmHg	83-108mmHg
	Lac	1.0mmol/L	0.5-1.7mmol/L
Coagulation	PT	11.6s	10.4-12.6s
	APTT	26.0s	23.3-32.5s
	D-Dimer	0.76mg/L	0-0.55mg/L
Inflammation markers	hsCRP	2.10mg/L	≤8.00mg/L
	TNF-α	86.7pg/ml	<8.1pg/ml
	ESR	3mm/h	0-15mm/h
HBV-DNA quantification	HBV-DNA	<1×10^3 copies/ml	<1×10^3 copies/ml

**Figure 2:**
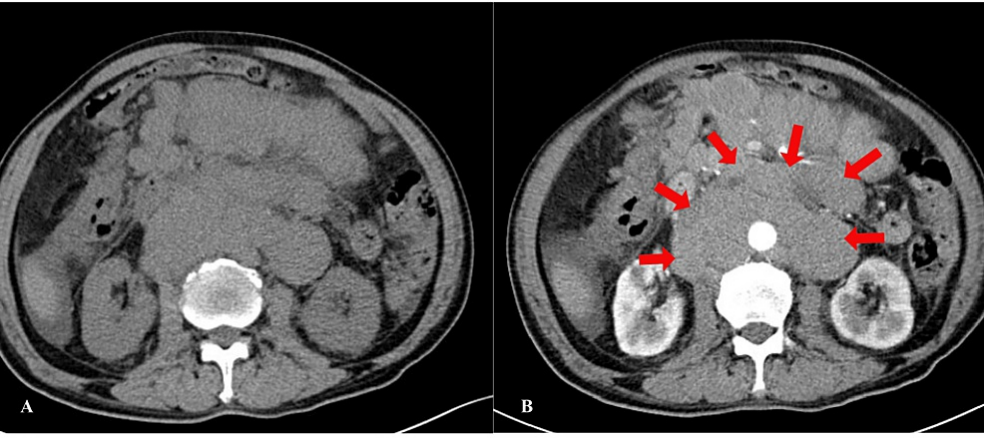
Enhanced CT of the abdomen Enhanced CT of the abdomen and pelvis reveals multiple masses in the abdominal and pelvic fat spaces, retroperitoneum, mesenteric area, and bilateral iliac vessels, possibly indicating manifestations of lymphoma. The abdominal aorta and its branches, portal system, and vena cava system are commonly affected, leading to vascular pushing displacement and compression stenosis.

**Figure 3:**
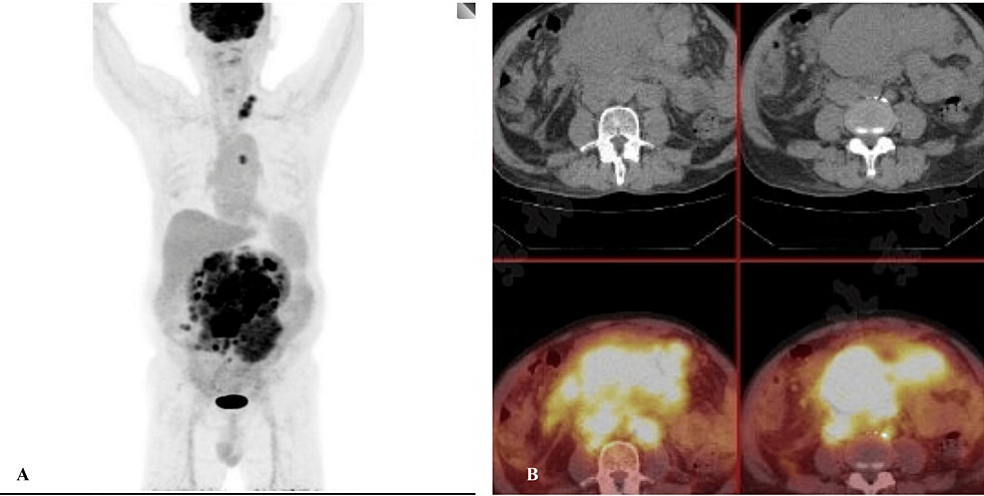
PET/CT PET/CT imaging displays numerous abnormal radioactive uptake masses and nodules located in the retroperitoneum, mesenteric area, bilateral common iliac vessels, and presacral area. PET/CT - positron emission tomography-computed tomography

**Figure 4:**
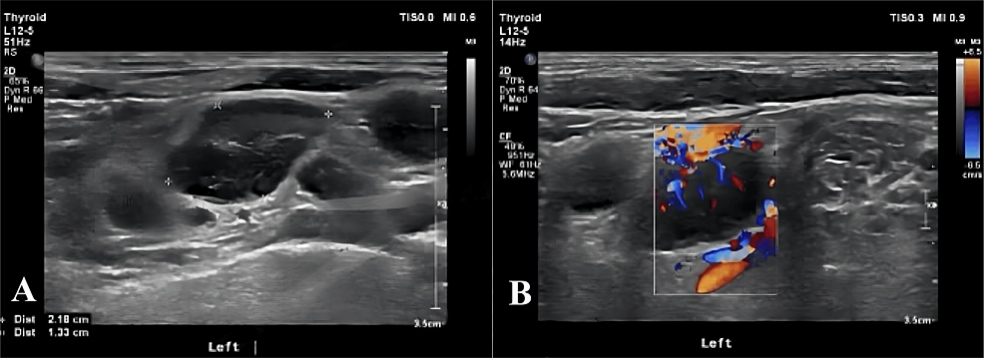
Ultrasound of lymph nodes Ultrasound of lymph nodes in the neck and supraclavicular fossa identified several hypoechoic lymph nodes, with the larger ones measuring 2.2×1.3cm and displaying an unclear corticomedullary boundary.

Imaging examinations revealed multiple spaces occupying the abdominal and pelvic fat spaces, retroperitoneum, mesenteric area, and bilateral iliac vessels, some of which were fused into a group with uniform enhancement. The abdominal aorta and branches, portal system, and vena cava were involved multiple times, with some blood vessels being pushed and displaced due to compression stenosis. The lesions were closely associated with the abdominal and pelvic intestines, although the division was not clear. Additionally, there was a small amount of fluid accumulation in the abdominal and pelvic cavity (Figure 2). Positron emission tomography-computed tomography (PET/CT) scan showed multiple abnormal radioactive uptake masses and nodules in various areas, some of which merged with each other and surrounded abdominal blood vessels (Figure 3). Ultrasound of lymph nodes in the neck and supraclavicular fossa identified several hypoechoic lymph nodes, with the larger ones measuring 2.2×1.3cm and displaying an unclear corticomedullary boundary. The CDFI revealed rich blood flow signals inside the lymph nodes (Figure 4). Echocardiography indicated no obvious abnormalities.

**Figure 5:**
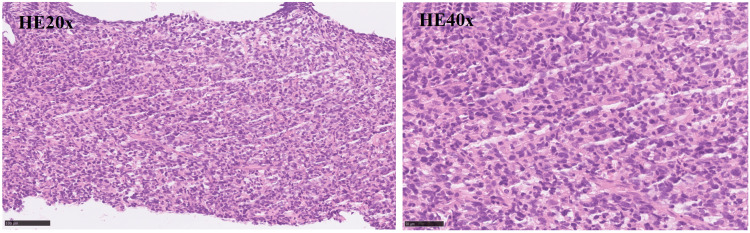
Pathology H&E 20x and H&E 40x: Pathological results indicate that the morphology is consistent with diffuse large B-cell lymphoma (germinal center origin)

**Figure 6:**
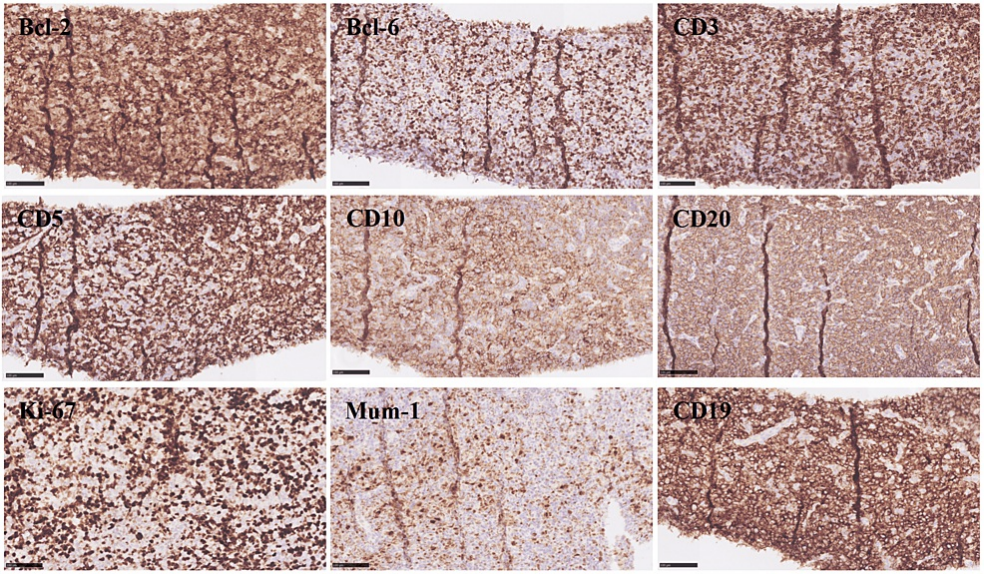
Immunohistochemistry Immunohistochemistry results showed positivity for Bcl-2 (90%), Bcl-6 (40%), CD3, CD5, CD10, CD20, CD30 (Ki-1), C-MYC, Ki-67 (50%), Mum-1 (30%), P53, and CD19, while CD21 was negative.

An abdominal mass puncture biopsy was performed, revealing pathology consistent with diffuse large B-cell lymphoma of germinal center origin (FIGURE 5). Immunohistochemistry results showed positivity for Bcl-2 (90%), Bcl-6 (40%), CD3, CD5, CD10, CD20, CD30 (Ki-1), C-MYC, Ki-67 (50%), Mum-1 (30%), P53, and CD19, while CD21 was negative (Figure 6). In situ hybridization results were negative for Epstein-Barr encoding region (EBER) in situ hybridization (ISH) and positive for the control. Morphological analysis of bone marrow cells indicated eosinophilia (16%). Bone marrow biopsy pathology revealed increased hematopoietic tissue with decreased adipose tissue and a normal granulocyte/erythroid ratio, along with elevated eosinophils and megakaryocytes. Immunohistochemical results included positivity for CD3, CD15, CD20, CD38, CD138, CD71, Ki-67 (90%), and MPO. Ascites cytology and pathology showed a predominance of T lymphocytes with no tumor cells. Immunohistochemistry results of ascites revealed positivity for CD3, CD2, CD5, CD7, CD4, CD8, CD10, Bcl-6, TIA-1, GranzymeB, CD30 (Ki-1), and negativity for CD56, CXCL-13, and PD-1.

The patient was diagnosed with DLBCL, Ann Arbor stage IIIA, based on imaging and pathological examination results. Treatment for the primary disease included abdominal puncture and drainage catheterization on April 30, 2024, with about 500ml of light yellow turbid ascites drained daily. The amount of ascite drainage decreased over time, leading to the removal of the abdominal drainage tube on May 7, 2024. Subsequently, on May 21, the patient was transferred to the hematology department for R-CHOP treatment. In terms of comorbidities, the patient's hypertension was managed with amlodipine 5mg once daily to maintain blood pressure within the range of 120-140/80-90mmHg. Additionally, for hepatitis B virus (HBV) infection, entecavir tablets at a dose of 0.5 mg once daily at 8 am were added to the treatment regimen.

## Discussion

The patient, an elderly male, presented with a subacute onset of disease. Three weeks prior, he experienced abdominal distension, which progressed to enlargement, hardening, and pain. In DLBCL patients, clinical manifestations can involve lymph nodes or extranodal sites. Typically, patients develop painless, progressively enlarging masses, often found in the neck or abdomen. Those with extranodal involvement may exhibit systemic symptoms and/or tumor lysis syndrome, along with palpable lymph node enlargement or masses in the neck, armpits, or groin [4]. Abdominal contrast-enhanced CT scans of abdominal/retroperitoneal lymphomas commonly show mass fusion with uniform density surrounding blood vessels, minimal necrosis, and mild to moderate enhancement post-contrast [5,6]. Elevated lactate dehydrogenase (LDH) levels and concurrent hepatitis B virus infection may be observed in laboratory tests [7,8,9]. Histopathological findings usually reveal diffuse proliferation of neoplastic large B lymphocytes and disruption of normal tissue structures [4]. The patient presented with a rapidly growing, painless abdominal mass, confirmed by physical examination. Abdominal contrast-enhanced CT scans revealed multiple masses in the abdominopelvic fat space, retroperitoneum, mesenteric area, and bilateral iliac vessels, showing partial fusion. The tumors were closely clustered, evenly enhanced, and consistent with lymphoma upon complete pathological examination, confirming diffuse large B-cell lymphoma of germinal center origin.

Lymphoma is a malignant tumor of the immune system that originates from lymph nodes or lymphoid tissue [1,2]. It is categorized into non-Hodgkin lymphoma (NHL) and Hodgkin lymphoma (HL), with B-cell lymphoma being more prevalent. The etiology and pathogenesis of diffuse large B-cell lymphoma (DLBCL) remain unclear and are associated with various factors such as heredity, gene mutations, immune dysregulation, and infections like HIV, hepatitis B virus, and hepatitis C virus [10]. Recent studies have highlighted a high HBV infection rate of 15.6% to 30.2% in DLBCL patients in China [7-9]. HBV infection can lead to abnormal liver function in DLBCL patients, significantly impacting their treatment and prognosis, and is closely linked to the development of lymphoma [11,12]. There are reports suggesting that HBV may trigger the onset of DLBCL by integrating into the host genome, resulting in oncogene overexpression or tumor suppressor gene downregulation in cells [13]. Chronic antigen stimulation and B lymphocyte proliferation can induce genetic abnormalities and double-stranded DNA fragmentation, promoting tumor growth [13]. Additionally, some studies propose that DLBCL may arise from the histological transformation of indolent lymphoma [14]. The patient has a significant increase in eosinophils. After reviewing the relevant literature [15], it is found that eosinophilia combined with diffuse large B-cell lymphoma is very rare. Based on the results of the bone marrow biopsy, which showed an increase in eosinophils (16%) and a significant number of eosinophils in the bone marrow pathology, it is considered that DLBCL has infiltrated the bone marrow, affecting the normal hematopoietic process and leading to an abnormal increase in the proportion of eosinophils [15].

Diffuse large B-cell lymphoma is the most common aggressive type of lymphoma, often characterized by painless progressive swelling of lymph nodes or localized masses. The majority of DLBCL cases are diagnosed in individuals aged 65 and older [16]. Approximately 40% of patients present with involvement of tissues outside of the lymph nodes, with bone marrow invasion seen in 10% to 20% of cases. DLBCL can originate in various tissues or organs, with the gastrointestinal tract being the most frequent location [1,4]. B symptoms, such as fever, weight loss, and night sweats, are present in around 30% of patients, and tumor lysis syndrome may also occur [17]. The occurrence of B symptoms is more common in patients who are positive for hepatitis B virus [17,18]. When DLBCL spreads and affects different organs, it can lead to high fever and symptoms related to those specific organs. Elevated levels of serum lactate dehydrogenase (LDH) and β-2-microglobulin are typically observed, and more than half of the patients are diagnosed with stage III-IV disease at the time of diagnosis [19,20].

The gold standard for diagnosing diffuse large B-cell lymphoma is through pathology, making biopsy of lesion tissue crucial for accurate diagnosis. As per the 2016 WHO revised DLBCL classification, DLBCL is characterized by a diffusely arranged large B-cell tumor exhibiting significant morphological heterogeneity [21]. The lymphoma cells are larger in size and diffusely distributed, with large and irregular nuclei containing rough chromatin and nucleoli [21,22]. Typically, these cells are centrally or eccentrically located, sometimes showing mitotic figures. They have rich cytoplasm with vacuoles or granules and are surrounded by small lymphocytes, histiocytes, and plasma cells, creating a messy background [21,22]. High mitotic figures indicate rapid cell proliferation [21,22]. The immunophenotype of DLBCL is closely linked to the cell's origin, which can be inferred by detecting specific markers on the tumor cell surface [23]. Germinal center B-cells, which are B-cells that activate and proliferate in lymph node germinal centers, can be identified by positive markers CD10, BCL-6, CD20, and CD19 of the germinal center B-cell-like (GCB) subtype [23,24]. In this case, the patient's Bcl-6 (40%+), CD10(+), CD20(+), CD19(+) expression suggests a germinal center origin.

The management of diffuse large B-cell lymphoma in elderly patients aged 60 years and above necessitates additional considerations compared to younger patients, largely due to the higher prevalence of comorbidities and poorer overall health status in this population. While R-CHOP remains the standard therapeutic approach for elderly DLBCL patients, alternative lower-intensity chemotherapy protocols or single-agent regimens may be necessary, depending on individual patient tolerance and comorbidities. Some research indicates that elderly patients could benefit from a shortened chemotherapy duration to minimize adverse effects [25]. Maintaining infection prevention measures and providing adequate nutritional support are crucial aspects of care for elderly DLBCL patients undergoing treatment [26]. In cases where elderly patients exhibit frailty and slow disease progression, particularly those with low tumor burden, a strategy of close disease monitoring and delaying treatment initiation until clear indications arise may be appropriate [25,26]. The management of DLBCL in elderly individuals should take into account the patient's overall health status, comorbidities, and personal preferences, emphasizing a personalized and cautious treatment approach that balances therapeutic efficacy with quality-of-life considerations.

## Conclusions

The symptoms of DLBCL in the early stages of the disease are often insidious and atypical. Similar to many other diseases, they are easily overlooked by patients and are frequently clinically disregarded and misdiagnosed. This article examines and explores the diagnosis and treatment process of this disease, aiming to enhance clinicians' knowledge of DLBCL.
